# 

**Published:** 1834-07-01

**Authors:** 


### CONTENTS

OF THE

### MEDICO-CHIRURGICAL REVIEW,

No. XLI. JULY 1, 1834.

[BEING No. I. OF A DECENNIAL SERIES.]

### REVIEWS.

I.

Pathological and Surgical Remarks on the Diseases of the Joints. By B. C. BRODIE, V.P.R.S. New Edition .. .. .. .. .. .. .. .. .. .. .. 1

II.

Illustrations of the Elementary Forms of Disease. By Dr. CARSWELL. [Fas. V.] 42

III.

Beobachtungen; or, Observations on Original Malformations, &c. .. .. .. .. 49

IV.

Consumption Curable; and the Manner in which Nature as well as Art Operates, &c. By Dr. RAMADGE .. .. .. .. .. .. .. .. .. .. .. .. .. 62

V.

A Practical Treatise on Diseases of the Joints. By W. J. WICKHAM, Esq. .. .. 68

VI.

A History of Egyptian Mummies, &c. &c. By T. J. PETTIGREW, Esq. .. .. .. 84

VII.

Hippopathology; or, Treatise on Diseases and Lameness of Horses. By W. PERCIVAL .. .. .. .. .. .. .. .. .. .. .. .. .. .. .. .. 88

VIII.

The Physiology, Pathology, and Treatment of Asphyxia. By Dr. J. P. KAY.. .. 92

IX.

MEDICAL BOTANY.

1. Hortus Medicus. By GEORGE GRAVES, M.D.. .. .. .. .. .. .. .. .. } 106

2. Medical Botany. By G. BURNETT, Esq. .. .. .. .. .. .. .. .. .. }

X.

Medica Sacra; or, Short Expositions of the most important Diseases mentioned in Scripture. By Dr. SHAPTER .. .. .. .. .. .. .. .. .. .. .. 109

XI.

Vindiciæ Medicæ; or, a Defence of the College of Physicians. By SIR GEORGE TUTHILL, M.D. .. .. .. .. .. .. .. .. .. .. .. .. .. .. .. 113

XII.

THE NERVES.

1. A Treatise on Diseases, &c. of the Nerves. By Mr. SWAN .. .. .. .. } 116
2. On the Reflex Function of the Medulla Oblongata. By Dr. M. HALL .. .. }
3. A New Exposition of the Functions of the Nerves. By J. W. EARLE, Esq. .. }

### PERISCOPE.

### PART I.

### Spirit of the English Periodicals, and Notices of English Medical Literature.

1. A new Synopsis of Nosology. Dr. Weatherhead .. .. .. .. .. .. .. .. 145
2. Anomalous Mental Affection .. .. .. .. .. .. .. .. .. .. .. .. 147
3. Fatal Effusion of Blood into the Pericardium .. .. .. .. .. .. .. .. 147
4. Treatment of Cholera in Ireland .. .. .. .. .. .. .. .. .. .. .. 150
5. On the Composition of the Pontine Marshes.. .. .. .. .. .. .. .. .. 152
6. Ligature of the Carotid Arteries for Head-aches, &c... .. .. .. .. .. .. 153
7. The Absentees; or, Blessings of Italy .. .. .. .. .. .. .. .. .. 154
8. The Sirocco. By Dr. Weatherhead .. .. .. .. .. .. .. .. .. .. 155
9. On Ulcers of the Legs. By Mr. Eccles .. .. .. .. .. .. .. .. .. .. 157
10. Temperance Societies—frightful Effects of Spirit-drinking in America .. .. 158
11. Provincial Hospitals—Memorial from the Bristol Infirmary to Mr. Warburton, 161
12. Editorial Report on Veratria .. .. .. .. .. .. .. .. .. .. .. .. 163
13. Mr. Cusack on certain Nervous Affections .. .. .. .. .. .. .. .. 165
14. Spurious Melanosis of the Lungs .. .. .. .. .. .. .. .. .. .. .. 166
15. Fracture of the Cervix Femoris, exterior to the Capsule .. .. .. .. .. 166
16. Ascites cured after repeated tappings .. .. .. .. .. .. .. .. .. .. 167
17. The Principles of Physiology applied to the Preservation of Health. By Dr. Combe .. .. .. .. .. .. .. .. .. .. .. .. .. .. .. .. 168
18. Reports from the Liverpool Medical Society .. .. .. .. .. .. .. .. 171
Separation of Symphysis Pubis—Croton Oil externally—Dreadful Accident—Anomalous Case—Hypertrophy of the Mamma—Permeability of the Optic Nerve by Light—Recumbent Treatment of Hernia—Double Vision—Pustular Eruption in Measles—Emetics in Apoplexy—Hibernation of Animals —Circulation in the Capillaries—Obscure and fatal Case—Hand-presentation in Labour .. .. .. .. .. .. .. .. .. .. .. .. .. .. 171 to 177
19. Transactions of the Provincial Medical and Surgical Association, Vol. II. .. 177
1. Case of Lithotomy by the Rectum .. .. .. .. .. .. .. .. .. .. 178
2. Case of Hydrophobia .. .. .. .. .. .. .. .. .. .. .. .. .. 179
3. Tuberculous Affection of the Kidney .. .. .. .. .. .. .. .. .. 180
4. Uterine Hydatids.. .. .. .. .. .. .. .. .. .. .. .. .. .. 182
5. Foreign Body found in the Heart .. .. .. .. .. .. .. .. .. .. 183
6. Reduction of Head of Humerus.. .. .. .. .. .. .. .. .. .. .. 184
20. Mr. Guthrie on Extraction of Cataract .. .. .. .. .. .. .. .. .. 184

ANATOMY AND PATHOLOGY.

21. Works of Dr. Hope—Dr. Carswell—Dr. Quain—Foreign Works .. .. .. 190

### PART II.

### Spirit of the Foreign Periodicals.

22. Vaccination in Italy .. .. .. .. .. .. .. .. .. .. .. .. .. .. 193

23. Dr. Hangstead on the Thymus Gland.. .. .. .. .. .. .. .. .. .. 195

24. Clinical Researches on the Medicinal Effects of Digitalis—from the Clinique of M. Andral—Hop. La Pitie .. .. .. .. .. .. .. .. .. .. .. 200

25. On the Influence of Gravity, and the depending Posture on the Circulation in Health and in Disease. By M. Gerdy .. 204

26. M. Velpeau's Remarks on certain Monstrosities .. 208

27. M. Majendie's Experiments on the Sounds of the Heart .. 208

28. Source of Animal Heat .. 209

29. Use of Steel in Menorrhagia .. 209

30. Remarks on Hooping-cough .. 210

31. Monomania .. 210

32. Treatment of Scrofula .. 211

33. Retroversio Uteri .. 212

34. Epistaxis and Hæmoptysis .. 212

35. Sympathy between the Cerebellum and the Testicles .. 212

36. Pomegranate Bark in Tænia .. 213

37. Effects of Mercury on Living Bodies .. 213

38. Illustrations of Sympathy in Diseases .. 214

39. Mercury in Rheumatism .. 214

40. Treatment of Epilepsy .. 214

41. Rupture of the Uterus .. 215

42. Lumbricus in a Wound .. 215

43. Simple Treatment of Facial Neuralgia .. 216

44. Iodine, Experiments on, in Scrofula .. 216

45. Somnolency lasting Four Months .. 217

46. Aconite in Neuralgia .. 221

Creosote in Ulcerations .. 221

47. Death from Cyanuret of Potass .. 222

48. On the Formation of the Epiploon in the Fœtus .. 222

49. Fatal Blow to Homoiopathy in Russia .. 223

50. Rupture of Vagina in Parturition .. 224

51. On the Pathology of the Cerebro-spinal Axis .. 225

52. Extensive Mesenteric Disease—Great Heat of Body .. 226

53. Indomitable Neuralgia of the Ulnar Nerve .. 227

54. Remarks on Neuralgia. By Scarpa .. 227

55. Complete Adhesion of a separated Finger .. 228

56. Suppression of Urine and Fæces for Eleven Years .. 228

57. Case of apparent Death lasting Three Weeks .. 229

58. Preservation of Leeches .. 229

59. Cold Injections into the Umbilical Cord to promote Separation of the Placenta, 230

60. Organic Disease of Lung from imperfect Respiration .. 230

61. On the Treatment of Porrigo .. 232

62. Hæmorrhagic Tendency in a Family .. 232

63. Ovaritis Puerperalis .. 232

64. Cyanosis on the Ninth Day .. 233

65. On the External Use of Croton Oil .. 235

66. On the Shortening of the Limb in Morbus Coxarius .. 238

67. On the Purulent Ophthalmia of Infants .. 239

68. On the Relation of the Cranium to the Organ of Hearing .. 240

### PART III.

### Clinical Review and Hospital Reports.

LOCK HOSPITAL.

69. Some Remarks on Venereal Condyloma, and on Venereal Warts. By HENRY J. JOHNSON, Esq. .. .. .. .. .. .. .. .. .. .. .. .. .. .. 241
1. On Condyloma .. .. .. .. .. .. .. .. .. .. .. .. .. 241
2. On Venereal Warts .. .. .. .. .. .. .. .. .. .. .. .. .. 247
3. On Chronic Inflammation of the Inner Prepuce .. .. .. .. .. .. 249

LONDON HOSPITAL.

70. Dislocation of the Head of the Femur into the Foramen Ovale .. .. .. .. 250

LONDON DISPENSARY.

71. Ascites cured after Paracentesis .. .. .. .. .. .. .. .. .. .. 251

DUMFRIES INFIRMARY.

72. Case of Empyema, with Operation. Dr. Grieve .. .. .. .. .. .. .. 253

GUY'S HOSPITAL.

73. Fracture of the Ribs and Sternum. (Bransby Cooper.) .. .. .. .. .. 255

TRALEE HOSPITAL.

74. Pneumo-Thorax, from Perforation .. .. .. .. .. .. .. .. .. .. 259

MEATH HOSPITAL.

75. Surgical Reports .. .. .. .. .. .. .. .. .. .. .. .. .. .. 260
1. Aneurism in Case of Necrosis .. .. .. .. .. .. .. .. .. .. 261
2. Fracture followed by Idiotcy .. .. .. .. .. .. .. .. .. .. 262
3. Bronchotomy.. .. .. .. .. .. .. .. .. .. .. .. .. .. 263
4. Diseased Lymphatics .. .. .. .. .. .. .. .. .. .. .. .. 264

GLASGOW ROYAL INFIRMARY.

76. Macfarlane's Clinical Report on Erysipelas .. .. .. .. .. .. .. 265

BIRMINGHAM EYE INFIRMARY.

77. Mr. Middlemore's Report .. .. .. .. .. .. .. .. .. .. .. .. 271
1. Changes in the Tarsal Cartilages .. .. .. .. .. .. .. .. .. 272
2. Staphyloma .. .. .. .. .. .. .. .. .. .. .. .. .. .. 272
3. Rheumatic Sclerotitis .. .. .. .. .. .. .. .. .. .. .. .. 273
4. Dislocation of the Lens .. .. .. .. .. .. .. .. .. .. .. 273
5. On the occasional Effects of Tumors in the Eyelids .. .. .. .. .. 275

MIDDLESEX HOSPITAL.

78. Wounds of the Knee-joint .. .. .. .. .. .. .. .. .. .. .. 275

79. Erysipelas .. .. .. .. .. .. .. .. .. .. .. .. .. .. 278

### Miscellanies.

80. St. Pancras Medical Association, with Remarks by the Editor .. .. .. .. 280
81. Spinal Deformities. Mr. Underwood .. .. .. .. .. .. .. .. .. 282
82. The Concours .. .. .. .. .. .. .. .. .. .. .. .. .. .. 283
83. Medical Reform .. .. .. .. .. .. .. .. .. .. .. .. .. .. 283
Bibliographical Record .. .. .. .. .. .. .. .. .. .. .. .. .. 285
EXTRA-LIMITES.—Mr. Arnott's Letter to the Editor .. .. .. .. .. .. .. 287

*** After the Bibliographical Record was closed, several Works came to hand, among which were Mr. Hemming's Translation of Mad. Boivin's Treatise on Diseases of the Uterus—Dr. Henderson's Translation of Raspail's Organic Chemistry—and Mr. Chitty's valuable Work on Medical Jurisprudence.

